# Unusual case of a gigantic bladder mimicking a large pelvic cystic mass

**DOI:** 10.1259/bjrcr.20220127

**Published:** 2023-01-14

**Authors:** Mohammad Khadeem Rumjaun, Sana Amjed, Muhammad Anas Ghazi, Jason Raw, Sohail Sabir

**Affiliations:** 1 Department of Medicine, Northern Care Alliance, Bury, Lancashire, United Kingdom; 2 Department of Radiology, Northern Care Alliance, Bury, Lancashire, United Kingdom

## Abstract

Pelvic masses are more common in females compared to the males. Bladder distension secondary to urinary retention can also mimic as a pelvic mass. However, it is rare to see chronic urinary retention with no clinical urinary symptoms. We present a case report of an elderly male who presented with abdominal pain and progressive worsening of breathing, along with abdominal distension. Initially, patient was thought to have a large cystic pelvic mass, causing bilateral renal hydronephrosis due to the ureteric compression. However, urinary cauterization drained 19,000 ml of the urine leading to not only resolution of the symptoms but also clinical improvement of the patient.

## Summary

Generally, pelvic masses are a lot more common in the female population as compared to the males. However, most common pelvic masses in males are usually cancers of the bowel, bladder, and prostate. Distended bladder secondary to urinary retention may also mimic a pelvic mass. This is the first case recorded locally where an enlarged prostate led to a chronic obstruction leaving up to 19,000 ml of urinary retention.

## Case presentation

**Figure 1.**
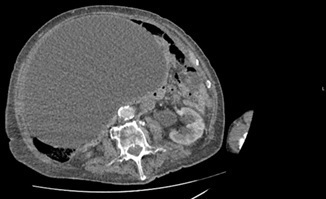
Axial CT image showing a massive homogeneous cystic mass. The wall of this large mass is thin, regular, and smooth and consistent with a fluid-filled mass.

**Figure 2.**
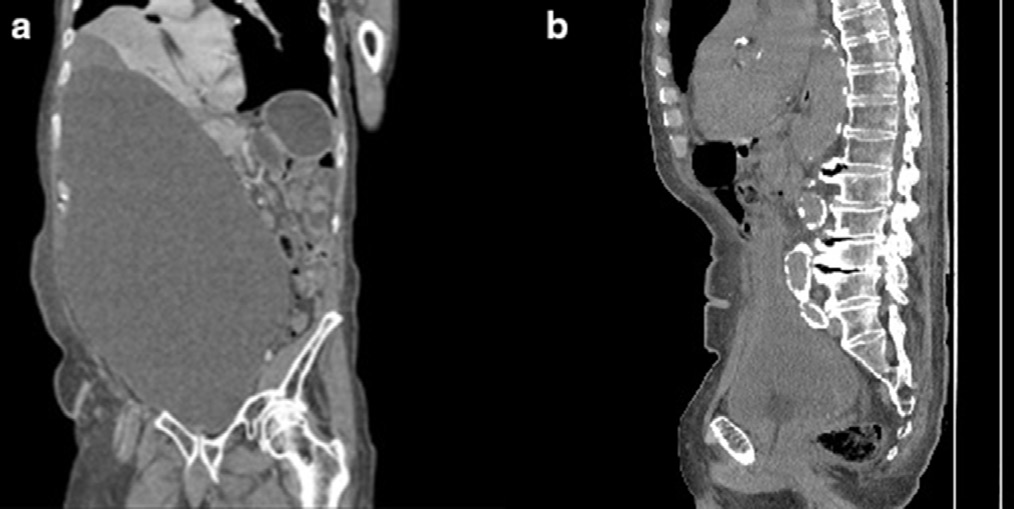
(a)Coronal CT image of abdomen and pelvis showed a better representation of the pelvic cystic mass. (**b**) Mid-sagittal view showing that the prostate is enlarged with a size of 4.3x 5.3 cm x 4.3 cm and a volume of 48.995 cm^3^.

**Figure 3.**
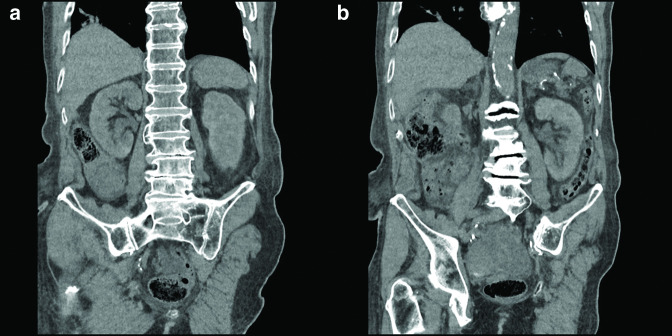
Coronal view of the right kidney (**a**) and the left kidney (**b**) before catheterization. The images show that the right kidney was normal and hydronephrosis of the left kidney.

We present a case of a 79-year-old male, who presented to the hospital with abdominal distension and pain, progressive worsening of shortness of breathing and inability to sleep for the past few weeks. Surprisingly, no urinary symptoms were reported by the patient. On examination, a large right hypochondrial mass extending to the hypogastrium was palpated along with generalized tenderness, but no features suggestive of peritonism were noted. Chest examination was clear with equal air entry at the bases bilaterally. An emergency CT scan of the abdomen and pelvis was requested to rule out any underlying malignancy which revealed a massive homogeneous cystic mass (measuring approximately 38*17*25 cm) in the right-side-abdomen extending from pelvis to compress the liver and cause raised right hemidiaphragm. (Figures 1 and 2) Overall appearance of this cyst was non-aggressive, but the origin was very difficult to determine partly because of the size. However, it was causing bilateral hydronephrosis, presumably from lower ureteric compression. The renal cortex enhanced normally.(Figure 3) The case was further discussed in the Radiology-Colorectal MDT, and it was suggested by the Radiologist that this cystic lesion might be a significantly distended bladder and advised for urinary catheterization to see if this cystic lesion gets decompressed. Otherwise, they also recommended interventional drainage of this humongous cystic lesion.

**Figure 4.**
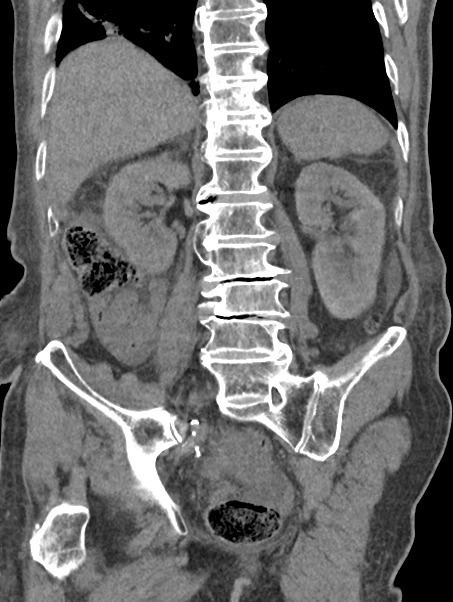
Coronal unenhanced CT image showing that post catheterisation there has been decompression of the grossly distended urinary bladder and a normal left kidney. The urinary bladder is hypertrophied and patulous secondary to chronic obstruction.

After catheterisation, 19,000 ml was drained in nearly 72 h resulting in significant improvement in patient’s clinical condition. Thereafter, a repeat CT scan was conducted which demonstrated the complete resolution of the gigantic cystic mass and hydronephrosis. (Figure 4)

### Treatment and follow up

The Urology team reviewed the patient in the outpatient clinic, and it was decided that patient will remain on long-term urinary catheter and was commenced on tamsulosin and finasteride. As part of his follow up plan, a trial without catheter was advised by the urologist to avoid any surgical intervention. Despite of multiple attempts, trial without catheter was failed in the community and patient was put on lifelong catheter to avoid similar episode of urinary retention in the future.

## Discussion

Urinary retention is a common presentation in the elderly population, especially amongst males. In general, within the next five years, 10% of males in their 70 s and 30% in their eighties will have urinary retention. The most known causes are Benign Prostatic Hyperplasia(BPH), bladder outlet obstruction such as urethral stricture, urinary calculus, neurogenic bladder, haematuria/clot retention, prostate and bladder neoplasm, and constipation.^1^ BPH accounts for at least 65% of the urinary retention in males and often preceded by lower urinary tract infection symptoms.^2^

Another point worth mentioning is that chronic urinary retention (CUR) is defined by the International Continence Society as ‘a non-painful bladder, which remains palpable or percussable even after the patient has passed urine.^3^ The interesting aspect of our case is the patient did not report any suprapubic pain, as you would expect in acute urinary retention, nor any other lower urinary tract symptoms until he presented with one of the largest bladders recorded on CT imaging in the world. Patient had been treated with recurrent UTI’s in the Primary Care setting, however, the possibility of the underlying etiologies, BPH amongst the most common, were never investigated.

## Conclusion

To conclude, while investigating cystic pelvic masses, the differential diagnosis of an overly distended bladder should be kept in mind, even in the absence of clinically correlating features, to avoid unnecessary invasive interventional procedures and their resulting complications. Also, elderly patients with frequent lower urinary tract symptoms, must be investigated with a detailed physical exam as well as urinalysis, measurement of the renal function, prostate-specific antigen and USS KUB to avoid complexities such as acute or chronic urinary retention. Timely recognition of BPH allows more effective management of its complication. Unfortunately, in this case, the patient has not been able to regain his normal bladder function and is unlikely to naturally void in the future.

## Learning points

- Chronic urinary retention can present without any suprapubic pain, especially in the elderly.
- Recurrent lower urinary tract symptoms in the elderly must be investigated with scrutiny and in the male population, ruling out BPH is important as it is very common.
- Diagnosing obstructive causes of urinary symptoms will be important in avoiding serious complications.
- CT images of one of the largest bladders ever recorded with a total amount of 19L of urine.

## References

[1] Selius BA, Subedi R . Urinary retention in adults: diagnosis and initial management. Am Fam Physician 2008; 77: 643–50. 18350762

[2] Muruganandham K, Dubey D, Kapoor R . Acute urinary retention in benign prostatic hyperplasia: risk factors and current management. Indian J Urol 2007; 23: 347–53. doi: 10.4103/0970-1591.35050 19718286 PMC2721562

[3] Negro CLA, Muir GH . Chronic urinary retention in men: how we define it, and how does it affect treatment outcome. BJU Int 2012; 110: 1590–94. doi: 10.1111/j.1464-410X.2012.11101.x 22452619

